# Site Readiness Framework to Improve Health System Preparedness for a Potential New Alzheimer’s Disease Treatment Paradigm

**DOI:** 10.14283/jpad.2022.32

**Published:** 2022-04-01

**Authors:** M. Anderson, N. Sathe, Cate Polacek, J. Vawter, T. Fritz, M. Mann, P. Hernandez, M. C. Nguyen, J. Thompson, J. Penderville, M. Arling, S. Safo, R. Christopher

**Affiliations:** 1 Premier Inc., Charlotte, NC USA; 2 RTI International, Research Triangle Park, USA; 3 Mount Sinai, New York, USA

**Keywords:** Alzheimer’s diease, mild cognitive impairment, new therapies, care pathways, site readiness framework

## Abstract

New therapies that address the underlying pathophysiology of Alzheimer’s Disease (AD), coupled with the growth of the AD population, will transform the AD care pathway and present significant challenges to health systems. We explored real-world challenges health systems may face in delivering potential new AD therapies with diverse stakeholders. Key challenges in care included integrating primary care providers into assessment and management, availability of memory care specialists, understanding payment and coverage issues and training mid-level providers to help coordinate care and serve as a shared resource across the system. This input informed a novel Site Readiness Framework for AD, comprising self-assessment exercises to identify health system capabilities and gaps and a framework of core strategies and responsive tools to help prepare to integrate new AD therapies. These resources may help health systems improve readiness to modify care pathways to integrate new therapies for AD.

## Introduction

**G**lobally, dementia is the seventh leading cause of death, yet less than 25% of people with dementia are actually diagnosed. In lower income countries, the percentage may be as low as 10% (1). Alzheimer’s disease (AD) is the most common cause of dementia, resulting in degeneration of brain regions related to learning, memory, and other cognitive domains that is ultimately fatal (2). This degeneration may begin up to 20 years before symptoms appear. Mild cognitive impairment (MCI) due to AD, the earliest symptomatic stage of AD (3), affects 15.8% of people 60 and older and 11.3% of people age 65 and older in the United States (US), and its prevalence is predicted to increase significantly as the population ages (4, 5). The prevalence of MCI ranges between 5.1% and 41% worldwide (6), and the burden of disease may double in G7 countries and nearly triple in G20 countries between 2015 and 2050.

AD is a leading cause of disability and death in older adults, and caring for individuals with AD leads to substantial costs for families and caregivers as well as society. Dementia caregivers face higher out-of-pocket costs than non-caregivers, both for the person affected with dementia and for their own care. Total annual dementia costs are estimated at $ 1.3 trillion worldwide (7) and $355 billion in the US, which does not include the costs of informal caregiving (4). A study from the Alzheimer’s Association (4) on potential cost savings of early diagnosis in the MCI phase rather than the dementia phase or not at all found that approximately $7 trillion could be saved in medical and long-term care costs due to a smaller spike in costs immediately before and after diagnosis of MCI, whereas diagnosis in the dementia phase has higher costs. In addition, costs would be lower for individuals with diagnosed and managed MCI and dementia compared with those without management. Treatments that prevent, cure, or slow disease progression may also contribute substantial savings to health care systems. Therefore, diagnosing AD early may result in benefits for patients and their family and caregivers as well as a potential cost savings in medical and long-term care costs (4).

Pathological hallmarks of AD, including amyloid plaques and neurofibrillary tangles, may accumulate decades before clinical symptoms appear, emphasizing the importance of early diagnosis (5, 8), as well as lifestyle changes and treatments that may help to slow disease progression and pathological accumulation (9). However, the path to an AD diagnosis is complex and variable. In the U.S. as well as in the rest of the world, most discussions about cognitive complaints originate in primary care, but primary care professionals often lack confidence in their ability to recognize neurocognitive disorders and frequently refer to specialists for cognitive assessment (1, 10). Wait time for appointments with a neurologist or for neuropsychological testing can be extensive (11). In addition, payment and coverage may be complicated (12, 13), and care may not be coordinated efficiently (14).

Historically, treatments for AD have only palliated symptoms, but new therapies in development may modify the underlying pathology of AD and offer patients the possibility of slowing cognitive decline. The increasing number of individuals with AD dementia, coupled with advances in biomarker-based pathological detection and the availability of therapies targeting underlying AD pathology, may help transform the way health systems identify and manage these patients (15).

To help health systems prepare to identify patients early in the course of AD and deliver new therapies, Premier Applied Sciences (PAS) and Biogen partnered to explore how new therapies may affect AD care, challenges to introducing new therapies, and approaches to help systems understand and improve their readiness to manage changes in AD care.

## Methods - Developing Understanding

**Figure 1:**
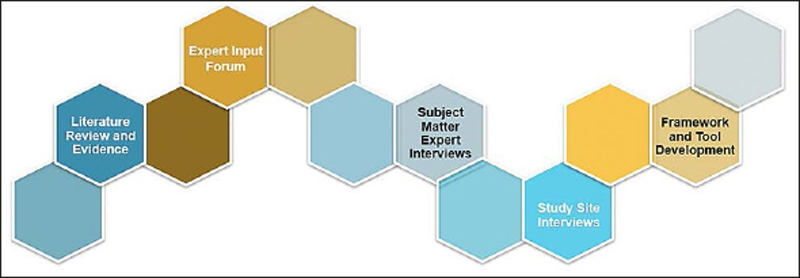
Project Approach

We sought to understand key challenges via a series of formative approaches. In July 2020, we conducted two focus groups with stakeholders in AD care including neurologists, geriatricians, psychiatrists, radiologists, care coordinators, infusion specialists and payor representatives. We also conducted individual interviews with several subject matter experts to provide further insights into focus group findings. In addition, we spoke with clinical and organizational leaders at four geographically dispersed health systems in the U.S. to understand how their unique system characteristics direct the ways they currently deliver care for cognitive concerns and how characteristics may direct their future delivery. Figure 1 outlines our approach. Table 1 outlines key results of this exploration.

**Table 1:** Key Formative Findings

**Area**	**Insights and Challenges**
Early identification	Providers strongly endorse a need for early identification of and engagement with the MCI population.
	Barriers to early identification include inadequate training of primary care professionals to identify cognitive impairment and a lack of economic incentives for primary care providers to conduct screening. Providers across the care continuum lack diagnostic confidence.
Access to specialty care	Relatively few neurologists specialize in memory issues, and their distribution across the U.S. health system is spotty. Efficient access to neurology care is an important challenge.
	Access to diagnostic and delivery tools like imaging and infusion centers may present roadblocks.
Evolving care pathway	Providers see providing broad access to care and understanding the care pathway for potential new therapies as key challenges to transforming AD care.
	An undefined and variable care pathway underscores the need for care coordination to ensure patients can navigate therapy and that connections among provider touchpoints are made efficiently.
System infrastructure	Health system structure in terms of affiliated sites, shared policy and technology infrastructure, and shared governance will influence how and where a system may choose to deliver new therapies.
Administrative impacts	Understanding the costs of potential new therapies, reimbursement logistics and potential payor requirements like prior authorization are important initial steps.

## Results

### Early Identification

Early identification of MCI is challenging as both patients and providers may consider changes to be related to normal aging and that little can be done to remedy deficits; patients and providers may also fear stigma associated with a dementia diagnosis (1, 16); While some patients self-refer for memory evaluation, many others may deny memory complaints, equate them with normal aging or may be unaware of the scope of the issue. Symptoms also vary among individuals, and manifestation may be subtle (17).

In addition, primary care providers feel particularly ill-equipped to screen for MCI or dementia. Cognitive testing in a typically short primary care visit seems infeasible, and providers may be unsure about how to proceed with patients with potential issues or lack confidence in their ability to interpret cognitive screening results (10, 16, 18, 19). Survey findings from Alzheimer’s Disease International indicate that primary care and general practitioners in other countries have similar challenges (1). Raising awareness on how to address cognitive concerns among patients and providers is an important step, and may include education, training and development or using new workflows. Fostering motivation to assess issues in primary care will be a key hurdle.

Those interviewed noted that family members often serve as the early warning system for cognitive complaints, and health systems will need to formalize their processes for obtaining caregiver input and fostering their participation in the patient journey. Similarly, although potential new therapies offer hope and the promise of slowing cognitive decline, therapies alone are not a magic bullet. Health systems must plan to support patients and their families and care partners, and consider equity and disparity issues in assessing patients and providing treatment (20). Providers will also need to understand how to manage patient expectations and communicate anticipated outcomes to patients among various populations.

### Access to Specialty Care

Patients with cognitive issues may be referred to neurologists for cognitive assessment; however, neurologist demand outpaces supply, both in the U.S. and worldwide (1, 15) with the greatest limitation on availability in rural regions (21–23). Moreover, neurology as a discipline is not monolithic. The number of neurologists equipped to deal with cognitive/memory issues is not well understood. Scarce supply and a growing population of aging individuals, plus the considerable amount of time required for full neuropsychological evaluation, lead to long wait times for specialist visits.

Among our sample of informants, wait times for cognitive evaluation or neuropsychiatric testing with a memory expert could be three months or longer (15), and full neuropsychological evaluation can take one to two hours. Given the projected size of the population who may develop AD, understanding the level of assessment required to determine need for further evaluation efficiently is critical, as well as who will administer the assessments. Technology may help to ease the burden of cognitive assessment and specialist access, as well (24). The COVID-19 pandemic jumpstarted telehealth integration, and some systems have mature telehealth or electronic consult processes in place, including telehealth approaches to cognitive assessment.

### Evolving Care Pathway

While the ultimate care pathway for new therapies will solidify with guidance for prescribing an agent, therapeutic initiation may utilize biomarker evaluation (tau/amyloid PET or lumbar puncture) to determine eligibility. Evolving care pathways will require care coordination to ensure that patients and care partners can manage care requirements.

Further, providers will need consistent standards for understanding amyloid positivity and interpreting biomarker tests and training in recognizing and managing potential complications of new therapies that target amyloid-beta; primary care and neurology providers also need education to understand treatment management and outcomes. Similarly, patients will need education to understand how new therapies work and approaches to measuring effectiveness, as therapies will not likely lead to improvements in cognitive function, but rather, will slow cognitive decline.

### System Infrastructure

Our informants felt that health systems understand the importance of collaborative, multidisciplinary care that incorporates advanced practice providers and care coordinators to deliver new therapies efficiently and in a patient-centered manner. Care coordination may positively affect utilization and quality of life in people with cognitive issues (25), but health systems in the U.S. and globally will need to integrate coordination into their routine processes and structure (15). In addition, our informants noted a need to share information widely and in multiple channels to catch providers broadly and in their preferred modality (email, learning management system, grand rounds, patient management conferences, etc.). Existing system-wide approaches to education and information sharing were variable.

Similarly, technology integration and centralizing processes to triage and prioritize requests is important for efficient and timely management of technology-enabled care. Distributed systems need to plan for elements such as sharing MRI images if systems lack a shared picture archiving and communication system and distributing expertise in reading images across the system. Health systems may also lack institution-wide approaches to plan for logistics and growth in demand of services like imaging and treatment administration.

### Cost Impacts

Insurance coverage and payment issues will likely not be clearly understood until new therapies begin to be dispensed. Systems will need to develop standardized approaches for understanding and managing payment processes, possible challenges for patients and families and equity issues. Coverage will likely also vary across carriers which may create additional challenges for health systems to keep up with requirements for varied policies. Informants particularly discussed potential coverage issues for biomarker testing that insurers may require to determine therapeutic eligibility (26).

## Solution Development

Using this input, we designed a large, multi-team project to draft a novel framework of strategies and tools designed to help health systems assess their readiness to adapt AD care for new therapies and to provide approaches to overcoming challenges to readiness. We used Premier’s 3Is Framework™ methodology to assess the steps needed to build a system approach to care and tactics to deliver care for the specific population. The “I”s stand for Identification, or activities needed to identify the appropriate patient population and gaps in system processes for acting on a new therapeutic area; Intervention, or the actions systems take to improve processes in the therapeutic area and steps providers should take at the point of care; and Interaction, which encompasses how providers across a system build referral patterns, advocate for resources and interact with patients around a new therapeutic area.

The Site Readiness for AD Framework provides a macro-level view of a therapeutic area using domains or key concepts to structure work in an area, and core strategies, the evidence-based processes and approaches to improvement that systems can adapt for their local contexts to evolve care and manage change. The Framework includes core domains comprising system-level preparedness, building system-wide competency in delivering new therapies, integrating care navigation and planning for access to therapies. Specific strategies in these domains can help sites consider process and logistical changes that may help their system prepare to deliver new therapies. Within the care navigation domain, for instance, strategies include developing individualized care plans that account for a patient’s specific care context and goals.

**Figure 2:**
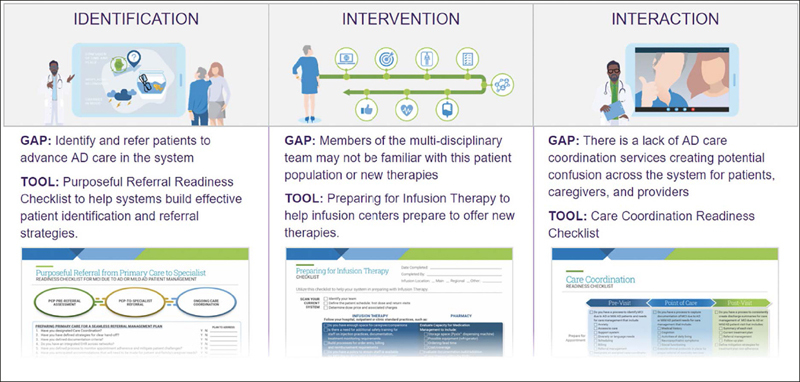
Framework Example

The Framework also includes specific actions or tools a system can use to help execute on strategies (Figure 2). We developed tools available in the Framework to address challenges raised in focus groups and discussions with health systems. For example, we created a “Purposeful Referral Checklist” to promote efficiency in the referral process and the likelihood that a patient with concerns “gets in the right door.” The checklist prompts providers to consider elements to facilitate seamless referral, including documentation criteria, hand-off procedures and specific care needs for the specialist provider to consider. These kinds of tools may help improve the completeness and appropriateness of referrals (27).

**Figure 3:**
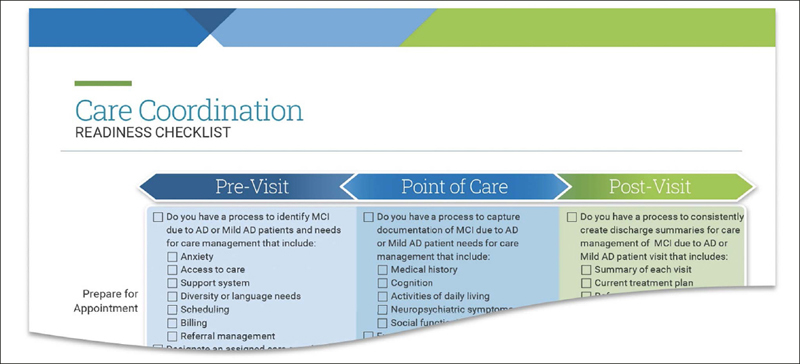
Care Coordination Readiness Checklist

Given the likely importance of care coordination to optimize delivering new therapies, tools also include a care coordination checklist. The checklist reviews core elements, such as patient and caregiver preparation, documentation guidelines and follow-up protocols, to consider in pre-visit, visit and post-visit settings. Providers and practices can use this tool (Figure 3) to foster coordinated care and potentially help mitigate caregiver stress (28).

**Figure 4:**
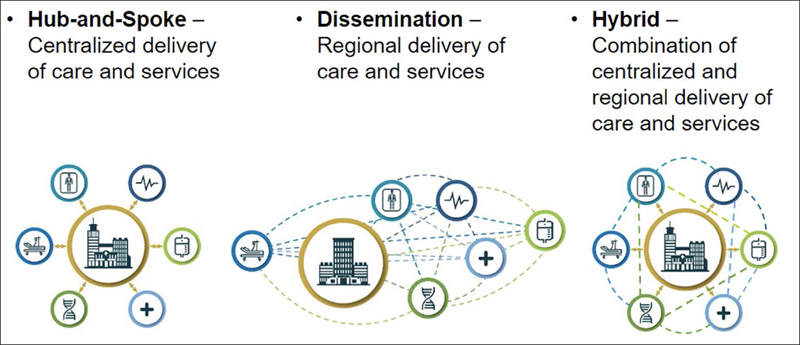
Archetype Models

We also developed a breakdown of characteristics to help explain how systems may choose to organize therapeutic delivery. Characteristics include elements such as the location, proximity and number of hospital sites, clinics and services, including specialized programs such as memory centers and diagnostic and ancillary services such as imaging and infusion. A system’s level of integration and standardization of infrastructure—in terms of policies, governance, technology and provision of education—as well as not-for-profit or profit and academic status—will influence delivering new therapies. These models, or archetypes, visualize care delivery and can help systems understand the effects of their physical and operational organization on access to care and potential trade-offs in situating the locus of care for conditions that require ongoing primary care and specialty care management (Figure 4).

In a hub-and-spoke model, most care is centralized at a main hospital and attached clinics—the hub (29). “Spoke” hospitals or clinics in the system typically offer limited services, and the system typically wholly owns individual ambulatory or acute sites. Primary care services are likely located off-site from the hub, but are often the first point of patient referral. Specialized services like infusion are localized at a main hospital which may allow patients to consolidate visits and plan for multiple appointments in one day. Hub-and-spoke institutions may have centralized technology, billing systems, policies and approaches to education, which may facilitate standardized processes across the system. Similarly, the hub-and-spoke model’s centralized organization may facilitate care coordination, although it may require some patients to travel to the hub for care. This model may also necessitate that patients balance considerations like in-network savings (e.g., using a local provider) and the convenience of hub-centralized care.

A more distributed, or disseminated, care model lacks a centralized hub and situates services at acute hospitals/clinics and ambulatory sites across a system’s network. Areas with broad geographically dispersed populations may use disseminated models, and disseminated systems may lack tightly centralized technology and management systems, policies and processes. Such locally developed management may make care coordination challenging. As with a hub-and-spoke model, patients may have to balance convenience (service location close to home) and network considerations. System organization may also reflect a hybrid or combination approach of hub-and-spoke and a disseminated model.

## Piloting Tools and Framework

We piloted tools with four health systems: two sites that used a disseminated model, one that used a hub-and-spoke model and one that used a hybrid model. Providers at the systems participated in brief phone discussions about their current processes for MCI and AD care, perceptions about changes in care with new therapies and opinions on drafts of the tools. System participants pilot-tested the Framework and tools in advance of availability of new therapies to inform how providers anticipate using the Framework and preliminary steps their organizations anticipate taking.

Participants pilot-testing the tools typically completed an assessment within the Framework to help prioritize areas for improvement. Most participants identified several areas in which they perceived deficits, but few respondents, even from the same system, identified the same areas for improvement. This variation in evaluation of system capabilities highlights a benefit of Framework use, namely, to understand multidisciplinary perspectives on system attributes. Because participants testing the Framework were doing so before the availability of new therapies, participants typically rated themselves lower in areas related to payer models, HIT-enabled AD care for patients, and setting goals related to new care models.

### Ongoing Challenges to AD Care

In alignment with subject matter experts, participants felt that neurology bandwidth will be a key limiting factor for access to new therapies, as well as understanding payment and coverage issues and authorization processes, which will evolve as payers and providers gain experience with a new therapy. In addition, sites will need to plan approaches to “connect the dots” between primary care providers, with whom patients may bring up cognitive complaints, and brain and memory specialists for assessment and potential prescribing of treatments.

### Planning for Memory Care Expansion

Participating systems are using the Framework to plan expansions of memory or brain health clinics. One site, which has a hub-and-spoke structure, is actively developing a centralized strategic roadmap for neurology care and incorporating Framework lessons. Another site with a disseminated configuration is negotiating internally and externally to develop focused care referral clinics across the system’s catchment area to provide cognitive assessment expertise. Participants also noted using the Framework to understand system attributes (e.g., online learning management system, infusion clinic network, specialist availability) that will facilitate therapeutic delivery across a system. Similarly, participants are considering telehealth approaches to formalize cognitive assessment and spread assessment capacity over the health system’s network. Participants also noted that they will engage in risk management discussions and plan related to new therapies, including strengthening referral relationships and assessing and understanding potential patient volumes to plan for multidisciplinary staffing approaches.

### Optimizing Communication Approaches

Participants recognize a need to plan proactive communication strategies addressing both patients and providers. Such communication will address the efficacy and safety of new therapies; given the novelty of new therapies, participants noted a need to understand trial data and expected outcomes associated with treatments. Participants are also planning clear, plain-language approaches to explain the mechanism of action of new therapies to patients and families, particularly as new therapies do not offer a cure for AD, but rather, target the progression of cognitive decline. Systems want to provide clear messaging about effects patients and families could expect, to appropriately manage those expectations and to consider how to address needs specific to older populations, as well as underrepresented populations. For instance, one participant noted that her system will need to prepare for managing continuity of therapy for “snowbird” patients who divide their time between states and sites of care.

Additionally, given the potential cost of new therapies and uncertain coverage restrictions or prerequisites, systems are thinking through access and equity issues and cost considerations. For example, participants want to understand potential trade-offs between the cost of therapy and potential savings anticipated with reduction in cognitive decline. Early diagnosis or delayed onset of dementia or functional decline is projected to result in substantial savings in costs for medical care and long-term care (4).

### Site Perceptions of the Framework and Tools

Participants uniformly endorsed the utility of the Framework for understanding readiness to deliver new therapies and agreed that project tools will help improve organizational readiness. Participants also felt they learned new information from the project and will share information learned with other colleagues. Participants endorsed the Framework’s emphasis on clinical and administrative champions to integrate and sustain process changes, and are incorporating Framework-informed thinking into strategic planning for adapting their AD care.

## Considerations for Practical Application

As new and potential therapies transform the care pathway for patients with MCI due to AD and mild AD, health systems that anticipate using these therapies will need to assess their current care pathways and determine how to refine them to ensure more timely access to diagnostic resources and treatments, especially for patients who are identified as exhibiting signs of early disease. To that end, health systems may want to consider the following elements to assess current practice and prioritize development goals:

•|Education for PCPs that includes conducting cognitive assessments in a standardized and timely manner for accurate diagnosis, appropriately referring patients to specialists, monitoring and managing side effects of treatment, and conducting effective and regular communication with a patient’s care team and caregivers/family.
•|Education for radiologists that includes interpreting potential novel safety findings, and for residents, fellows, nurses, and licensed social workers that includes taking patient histories and conducting cognitive assessments and other tests.
•|An interdisciplinary care team to help manage assessment, diagnosis, treatment, and ongoing care to accommodate treatment approaches that typically include a pharmaceutical intervention, cognitive therapy, and social services engagement.
•|A memory care clinic as a central point of contact for the interdisciplinary care team that uses electronic health records to initiate and facilitate communication between providers and to coordinate patient care and resources.
•|If establishing a memory care clinic is not feasible either immediately or long-term, then a system may want to consider designating care coordinators to serve as central points across the AD patient care pathway to facilitate care by managing communication across providers, managing patient and caregiver expectations, making appointment reminder calls, providing access to educational materials and support groups for patients and caregivers, ensuring understanding of insurance coverage, and scheduling follow-up appointments.
•|Processes and documentation for reviewing new therapies for formulary consideration as well as what information insurance companies will require for coverage.

## Limitations

These findings should be interpreted in light of potential limitations. We interviewed a small number of participants, although the sample size aligns with exploratory qualitative research methods (30–32). The small participant number is mitigated by the variety of roles and the breadth of perspectives represented in the interviews. The persons interviewed were also all U.S.-based providers, so generalizability of findings to the broader global population of providers who treat MCI and AD is limited, although literature and reporting from organizations such as Alzheimer’s Disease International indicate that challenges and patient pathways in the care continuum in other countries are similar (1) and align with our findings in this project. Finally, the first new therapy hadn’t been approved yet in 2020 when this project was conducted, and other therapies either were or still are in clinical trials or in the FDA submission process for approval. Therefore, provider opinions and perspectives in the interviews on the potential impact these therapies may have on the U.S. healthcare system were somewhat speculative. Two years on, with the first therapy now available and others pending FDA approval, we anticipate any new therapies would still require similar considerations as expressed in this paper.

## Conclusions

New therapies for AD offer hope to a large and growing patient population, and will require new modes of delivery for AD medications. Therapies introduce challenges for health systems in terms of understanding and managing the logistics of patient identification, intervening to ensure efficient therapeutic delivery and continuing interaction to monitor patient needs and ongoing health system adaptations. The Site Readiness for AD Framework offers a comprehensive approach to allow health systems to consider changes to processes and operations to transform their AD care. System archetypes may help hospital leaders understand where to position new therapies optimally within their network structures. Together, the Framework and system archetypes encompass input from broadly representative clinicians providing care for MCI and AD patients and from health systems participating in reviewing the Framework. The Framework’s structured approach to thinking through care and administrative needs, readiness strategies and tools to help with preparing to modify care provide a useful roadmap for health systems to follow in integrating new therapies.
